# Comparison of clinical and biological data between septic arthritis of the hip and those of the knee caused by *Kingella kingae*

**DOI:** 10.1097/BPB.0000000000001275

**Published:** 2025-07-25

**Authors:** Giacomo De Marco, Blaise Cochard, Ardian Ramadani, Elvin Gurbanov, Giorgio Di Laura Frattura, Anne Tabard-Fougère, Nathaly Gavira, Christina Steiger, Romain Dayer, Dimitri Ceroni

**Affiliations:** Department of Women, Children and Adolescents, Pediatric Orthopedic Unit, Pediatric Surgery Service, Geneva University Hospital, Geneva, Switzerland

**Keywords:** C-reactive protein, hip, joint, *Kingella kingae*, knee, pediatric, septic arthritis, temperature

## Abstract

*Kingella kingae* is the leading cause of osteoarticular infections in children under 4 years, with septic arthritis (SA) being the most common manifestation. The present study aimed to define the clinical and biological characteristics of SA of the hip and of the knee caused by *K. kingae*, and, secondarily, identify whether there were significant differences between them; this with the objective to assess if different possible strategies of diagnosis and treatment could be applied to different joints. Medical records of 100 children (50 hips, 50 knees) with confirmed *K. kingae* SA were analyzed, including sex, age, temperature, white blood cell (WBC) count, platelet count, C-reactive protein (CRP), and erythrocyte sedimentation rate (ESR). Children with hip SA had a significantly higher proportion of fever ≥38.5 °C (42% vs. 14%, *P* < 0.05) and higher median CRP levels (32.0 vs. 21.0 mg/L, *P* < 0.05) compared with those with knee SA. No significant differences were found in WBC count, ESR, or platelet count. These findings suggest that hip SA presents with more pronounced systemic inflammation (higher fever and CRP) than knee SA. Further research is needed to assess whether SA in other joints (e.g. ankle, wrist, and shoulder) also exhibits distinct clinical and biological patterns. This study highlights potential differences in *K. kingae* SA presentation based on joint involvement, which may influence clinical management. Further studies seem essential to understand whether SA affecting other joints (e.g. ankle, wrist, and shoulder) also results in specific clinical and biological presentations.

## Introduction

Kingella *kingae* is a Gram-negative anaerobic facultative β-hemolytic coccobacillus first described in 1960 [1]. This bacterium has a relatively slow growth, and it is difficult to identify in normal cultures [2]. As shown in previous works [3], it could be found in the oropharyngeal flora of infants between 6 months and 5 years old where it can grow because of their weak immune system in this age range [1].

Over the last 20 years, the consistent use of nucleic acid amplification tests has resulted in the recognition that *K. kingae* is the primary agent of joint and bone infections in children from 6 to 48 months old [4–7]. It is now well established that osteoarticular infections (OAIs) caused by *K. kingae*, most commonly septic arthritis (SA), tend to involve the lower limbs and usually the knees [7–11].

The clinical and laboratory presentations of children with *K. kingae* SA (and all OAIs caused by this germ) are subtle, atypical, and characterized by a mild clinical picture with moderate local and systemic inflammation markers, thus requiring a high index of attention [4–7,11–14]. Furthermore, the synovial white blood cell (WBC) counts of children with culture-proven arthritis caused by *K. kingae* show values of >50 000/ml in only a quarter of cases, and Gram stain testing usually remains negative [13,14]. All these issues make *K. kingae* SA difficult to diagnose using unambiguous clinical and biological criteria.

Given the heterogeneous presentation of *K. kingae* SA, clinicians can legitimately ask themselves whether the type of joint might play a determining role in the infection’s clinical and paraclinical manifestations. There is currently no evidence to assert that the clinical and biological presentation of *K. kingae* SA has the same characteristics in the hip and knee.

The present study’s primary objective was to define the clinical and biological aspects of *K. kingae* SA of the hip and the knee, and secondarily, to assess whether there were significant differences in the disease’s presentation depending on its localization, with the aim to assess if different diagnostic and treatment strategies could be applied to different joints.

## Materials and methods

After approval by Geneva Children’s Hospital’s Ethics Review Committee, we retrospectively reviewed the medical charts of every child aged from 0 to 15 years old admitted to our institution with confirmed SA between January 2007 (when the use of a routine molecular detection method for *K. kingae* began in our institution) and December 2023. We established two separate subgroups of 50 children with *K. kingae* SA of the hip and 50 with *K. kingae* SA of the knee.

The main reason for admission was refusal to bear weight or limping. These symptoms were accompanied by fever (>38 °C) in 27 (54%) cases in the hip group and 18 (36%) cases in the knee group.

We defined a confirmed *K. kingae* SA as cases with either positive cultures (blood or joint fluid) or a positive PCR assay for *K. kingae* (blood or joint fluid), or both.

The following data were noted for each patient: sex, age, temperature at admission, weight-bearing status, WBC count, platelet count, CRP value, erythrocyte sedimentation rate (ESR), and detailed results of the bacteriological investigations. The laboratory values were the first values encountered upon the admission to the hospital. The following cutoff values were applied for both groups: fever defined as a body temperature of ≥38 °C; WBC > 17 000/mm^3^ for children less than 4 years old and >12 000/mm^3^ for ≥4 years old; CRP > 10 mg/L; and ESR > 20 mm/h [15,16]. The following exclusion criteria were used to avoid information bias associated with incomplete data analysis and selection bias associated with the inclusion of patients with presumptive and inconsistent diagnoses: (1) no bacteriological diagnosis was available, (2) no laboratory data were available, and (3) no antibiotics were administered.

### Microbiological workup

Blood cultures are used systematically to isolate the microorganisms responsible for SA. A fluorescence-quenching-based oxygen sensor system (BACTEC 9000, Becton Dickinson, Sparks, Maryland, USA) was used before 2009, and an automated blood culture system (BD BACTEC FX, Becton Dickinson, Sparks, Maryland, USA) was used after that date. Joint fluid was analyzed using Gram staining, cell count, and immediate inoculation onto Columbia blood agar (incubated under anaerobic conditions), CDC anaerobe 5% sheep blood agar (incubated under anaerobic conditions), chocolate agar (incubated in a CO_2_-enriched atmosphere), and brain–heart broth. Total incubation time was 10 days. Two PCR assays were also used for bacterial identification when standard cultures were negative. Initial specimens (100–200 µl) were stored at −80 °C until being processed for DNA extraction. A universal, broad-range PCR amplification of the *16S rRNA* gene was performed using BAK11w, BAK2, and BAK533r primers (Eurogentec, Seraing, Belgium). Real-time PCR assays targeting the RtxA and RtxB cytotoxins expressed by *K. kingae* were performed from 2007 onward. Since September 2009, an oropharyngeal swab to detect *K. kingae* using a PCR has been available for children aged 6–48 months old, improving our diagnostic approach. When *K. kingae* is found in the oropharyngeal cavity using this simple technique, there is strong evidence that this microorganism is responsible for the OAI [3,11]

### Statistical analysis

The characteristics of patients with *K. kingae* SA were analyzed in two subgroups (SA of the hip and SA of the knee). Clinical manifestations and laboratory test results were expressed as means, SDs, and ranges [min–max]. The normality of the distribution was evaluated using a normal Q–Q plot and the Shapiro–Wilk test. Comparisons between patients with *K. kingae* SA of the hip and those with *K. kingae* SA of the knee were performed using Student’s *t* tests for continuous outcomes with normal distribution reported with mean (SD), unpaired Mann–Whitney *U* test for continuous outcomes with non-normal distributions reported with median (interquartile range), and a Pearson’s chi-squared test for dichotomous outcomes reported with *n* (%). Statistical analyses were performed using R software, v.4.2.2 (R Foundation for Statistical Computing, Vienna, Austria), with the RStudio interface (RStudio Team 2016, RStudio, Inc., Boston, MA, USA). Statistical significance was set at *P* < 0.05. Cohen’s effect size and two-sided 95% confidence intervals were also reported.

## Results

We collected 100 confirmed cases of *K. kingae* OAI (50 hips and 50 knees) from 2007 to 2023, including 59 boys and 41 girls (Table 1). *K. kingae* was detected using either direct culture, PCR amplification, or both. Ninety-nine cases of *K. kingae* were identified using a joint fluid PCR assay specific for *K. kingae*, one case was identified in a joint fluid culture, and zero cases were identified using blood PCR. The mean age of children with a confirmed SA caused by *K. kingae* was 16.7 ± 9.1 months, ranging from 4 to 66 months old (Table 1). One confirmed SA caused by *K. kingae* occurred in a patient < 6 months old and one in a child > 4 years old. The main reason that brought patients with *K. kingae* SA of the hip to the emergency department (ED) was a refusal to bear weight on the affected limb (30 patients of 50, 60%). On the contrary, the main reason bringing patients with *K. kingae* SA of the knee to the ED was a limp on the affected limb (27 patients, 50%).

**Table 1:** Epidemiological data of children with septic arthritis (SA) of the hip and the knee caused by *Kingella kingae*

	*K. kingae* SA of the hip (*n* = 50)	*K. kingae* SA of the knee (*n* = 50)
Age, mean (SD) [min–max], months	15.8 (8.4) [4.0–45.0]	17.6 (9.8) [6.0–66.0]
Gender	30 males/20 females	29 males/21 females
Age repartition		
0–12 months	24 (48%)	15 (30%)
13–24 months	19 (38%)	27 (54%)
25–36 months	5 (10%)	7 (14%)
37–48 months	2 (4%)	0 (0%)
>48 months	0 (0%)	1 (2%)

### Kingella *kingae* septic arthritis of the hip

We observed that 23 patients (46%) with a confirmed *K. kingae* SA of the hip were afebrile (temperature < 38 °C) at admission (Table 1), but most had presented a peak fever >38 °C before admission. Among feverish children, 21 (42%) had a temperature ≥ 38.5 °C. WBC count was considered elevated in 32 cases (64%) (>12 000/ml), and no left deviation was encountered. When the adjusted cutoff values were applied (>17 000/ml in children < 48 months old), WBC counts were abnormal in nine cases (18%). CRP was elevated (>10 mg/L) in 42 patients (84%). ESR was elevated (>20 mm/h) in 79% (*n* = 22/28 with available data) of cases and was >40 mm/h in 50% (*n* = 14/28) of children. Finally, platelet counts were abnormal (>400 000/ml) in 68% of cases (*n* = 25/37 with available data).

### *Kingella kingae* septic arthritis of the knee

We noted that 32 patients (64%) with confirmed *K. kingae* SA of the knee were afebrile (temperature < 38 °C) at admission, but most had presented a peak fever >38 °C before admission. Among feverish children, only seven (14%) had a temperature ≥ 38.5 °C. WBC count was considered elevated in 26 patients (52%) (>12 000/ml), and no left shift was noted. When the adjusted age cutoff values were applied (>17 000/ml in children < 48 months old), WBC counts were abnormal in five children on 49 (10%). CRP was elevated (>10 mg/L) in 40 patients (80%). ESR was elevated (>20 mm/h) in 71% (30/42 with available data) of cases and was >40 mm/h in 33% (14/42) of children. Finally, platelet counts were abnormal (>400 000/ml) in 52% of cases (25/48 with available data).

### Comparison of the demographic, clinical, and biological characteristics of patients with septic arthritis of the hip and septic arthritis of the knee caused by *Kingella kingae*

**Fig. 1:**
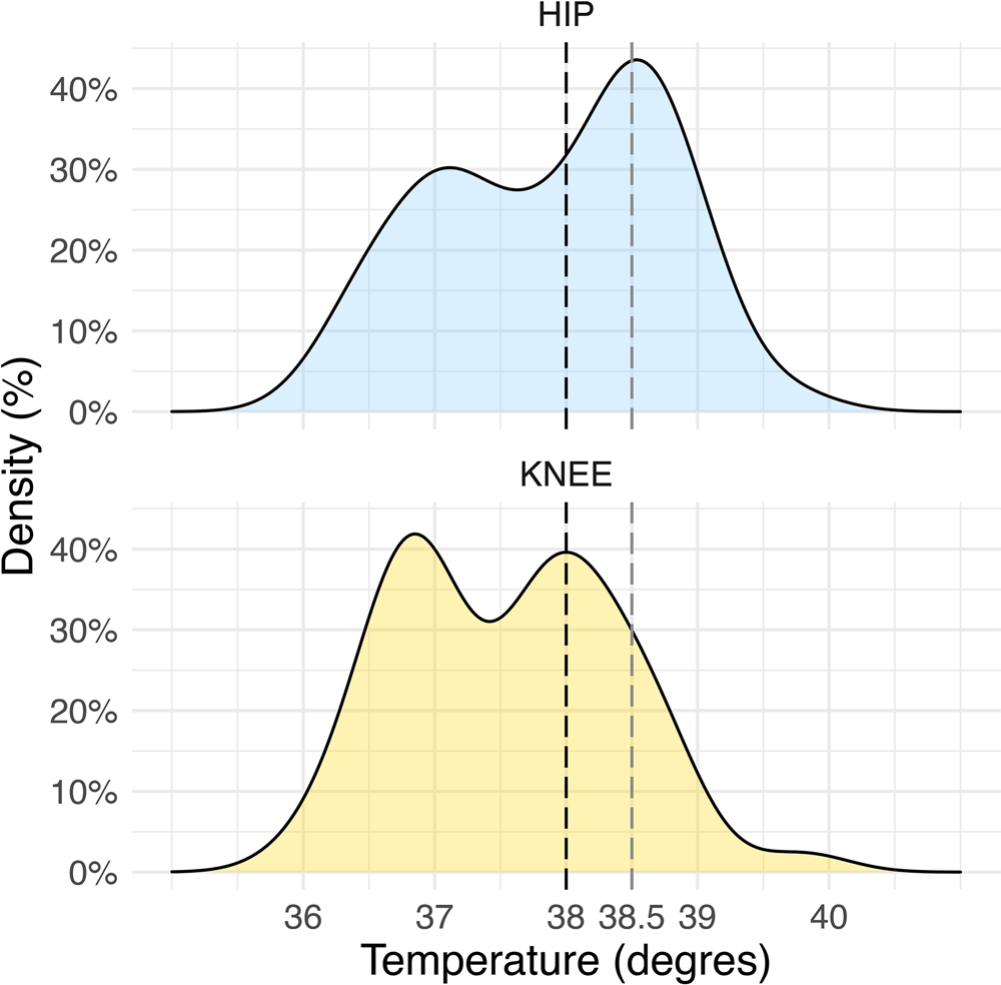
Density curve of the temperature (degrees) at the entry for patients with septic arthritis (SA) caused by *Kingella kingae* hip (blue) and knee (orange) arthritis.

**Fig. 2:**
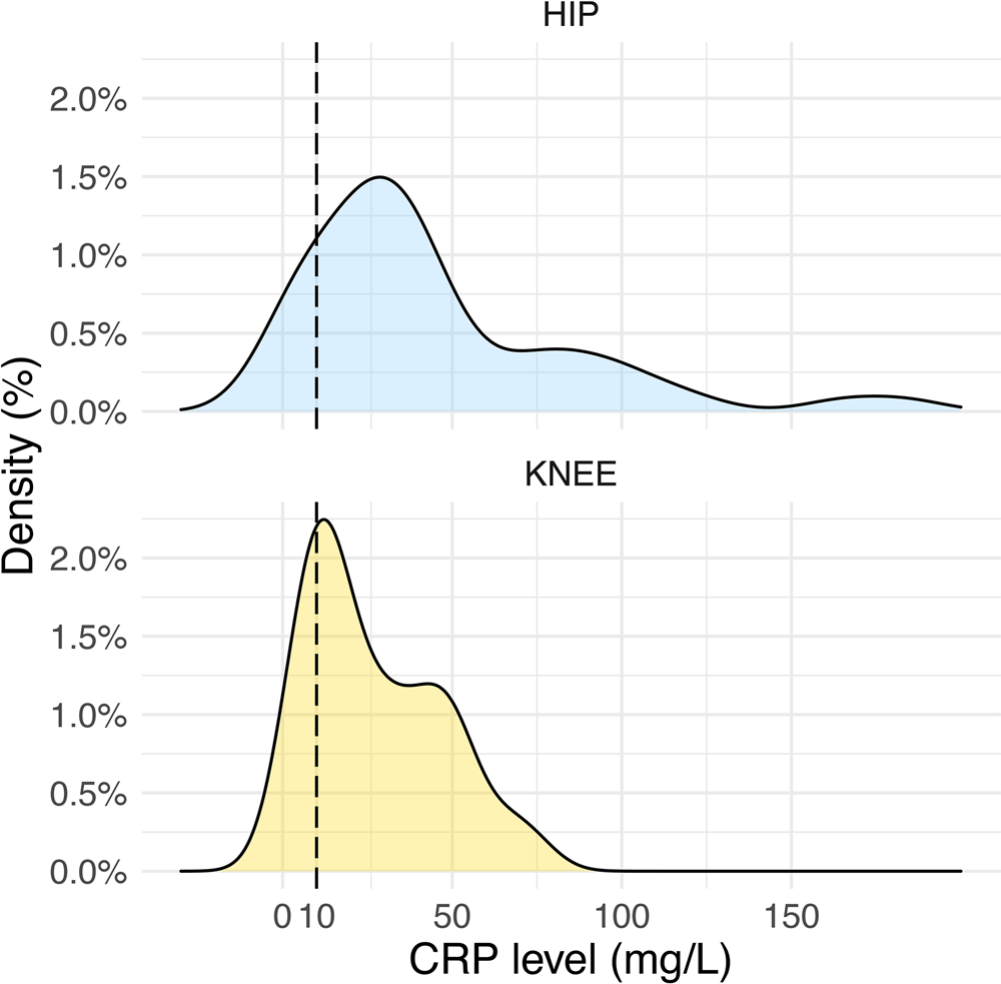
Density curve of the CRP level (mg/L) for patients with septic arthritis (SA) caused by *Kingella kingae* hip (blue) and knee (orange) arthritis. CRP, C-reactive protein.

**Fig. 3:**
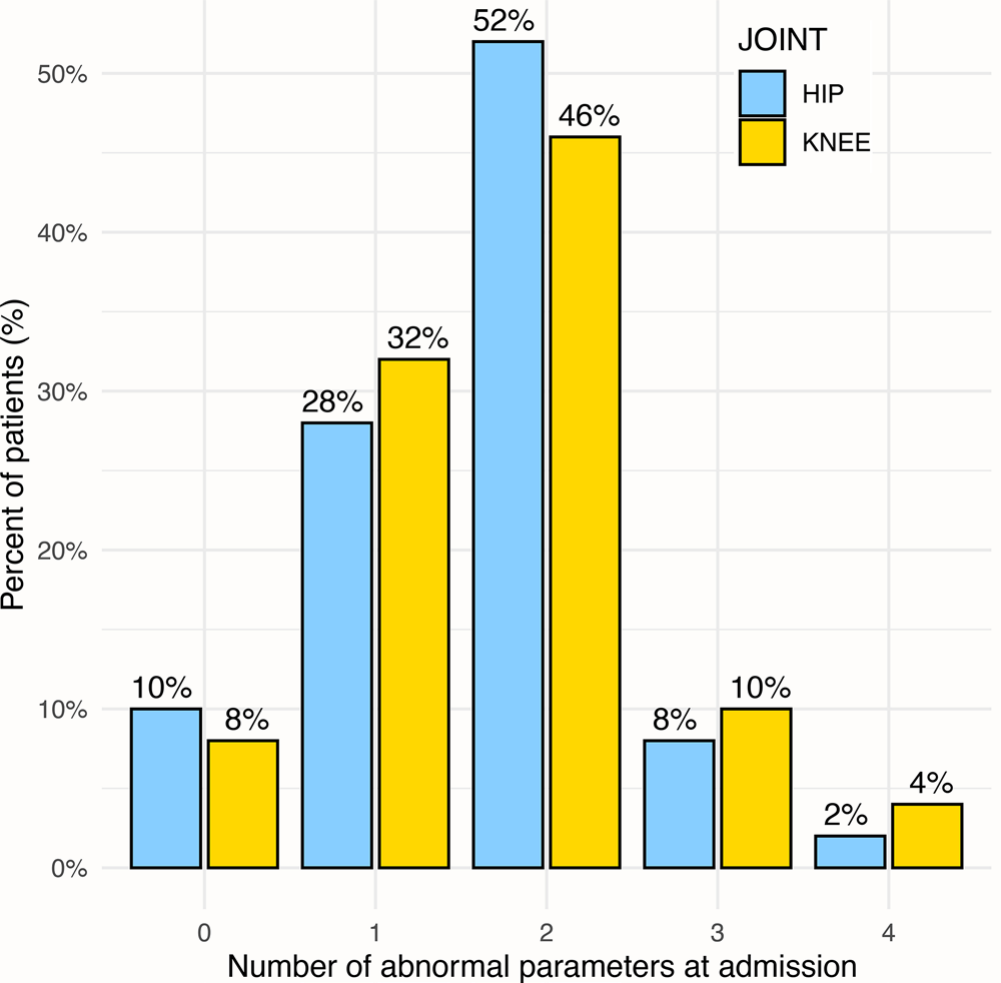
Number of abnormal values present at admission in percent for the four usual infection predictors (temperature ≥ 38 °C; WBC ≥ 17 000/mm^3^ for children less than 4 years old and ≥12 000/mm^3^ for those ≥ 4 years old; ESR ≥ 20 mm/h; and CRP ≥ 10 mg/L) for patients with septic arthritis (SA) caused by the *Kingella kingae* in hip (blue) and knee (orange) arthritis. CRP, C-reactive protein; ESR, erythrocyte sedimentation rate; WBC, white blood cell.

There was no statistically significant difference in the age distributions at admission between the two subgroups of SA caused by *K. kingae*. We nevertheless noted a significantly higher temperature (*P* = 0.034) among children with SA of the hip compared with those with SA of the knee (Table 2); moreover, the proportion of children with a temperature ≥ 38.5 °C was significantly higher (*P* = 0.004) among children with SA of the hip (42%) than among those with SA of the knee (14%) (Fig. 1 and Table 3). Concerning inflammatory markers, there were no significant differences between the subgroups regarding WBC count, ESR, and platelet count (Tables 2 and 3). There, however, was a significant difference in CRP values (Fig. 2 and Table 2), with children with *K. kingae* SA of the hip having higher values (*P* = 0.048). As reported in Fig. 3, with regard to the ‘classical’ combined infection predictors (temperature ≥ 38 °C; WBC ≥ 17 000/mm^3^ for children < 4 years old and ≥12 000/mm^3^ for ≥4 years old; ESR ≥ 20 mm/h; and CRP ≥ 10 mg/L), the distribution was not significantly different between SA of the hip and the knee. The majority of cases encountered two or less parameters, with, respectively, 90% of the hips and 86% of the knees.

**Table 2:** Comparison of the clinical and biological data between the two subgroups (hip vs. knee) of septic arthritis caused by *Kingella kingae*

	Hip (*n* = 50)	Knee (*n* = 50)	*P*	ES	95% CI
Temperature, °C	38.0 (37.0–38.5)	37.6 (36.9–38.1)	0.034*	0.183	0.1 to 0.7
WBC count, 1000/mm^3^	13.7 (11.3–15.9)	12.4 (10.1–14.5)	0.161	0.010	−0.4 to 2.4
CRP level, mg/L	32.0 (18.3–56.8)	21.0 (12.0–43.0)	0.048*	0.166	0.2 to 20.0
ESR, mm/h^a^	40.5 (24.8–54.3)	31.0 (20.0–42.8)	0.168	0.096	−3.0 to 18.0
Platelet count, 1000/mm^3^^b^	420 (339–541)	411 (332–521)	0.395	0.027	−27 to 87

Results are presented as median (IQR). Group comparisons are made with a Mann–Whitney *U* test.

CI, confidence interval; CRP, C-reactive protein; ES, effect size; ESR, erythrocyte sedimentation rate; IQR, interquartile range; WBC, white blood cell.

a ESR data were available for *n* = 28 hip and *n* = 42 knee.

b Platelet count data were available for *n* = 37 hip and *n* = 48 knee.

* Significance was set at *P* < 0.05.

**Table 3:** Comparison of the clinical and biological criteria at admission between the two subgroups (hip vs. knee) of septic arthritis caused by *Kingella kingae*

	Hip (*n* = 50)	Knee (*n* = 50)	*P*	ES	95% CI
Temperature < 38 °C	23 (46%)	32 (64%)	0.108	0.259	−39 to 3%
Temperature ≥ 38.5 °C	21 (42%)	7 (14%)	0.004*	0.838	9 to 47%
WBC count > 12 000/mm^3^	32 (64%)	26 (52%)	0.311	0.103	−9 to 33%
WBC count > 17 000/mm^3^^a^	9 (18%)	5 (10%)	0.410	0.068	−8 to 23%
CRP level > 10 mg/L	42 (84%)	40 (80%)	0.795	0.007	−13 to 21%
ESR > 20 mm/h^b^	22 (79%)	30 (71%)	0.696	0.018	−16 to 31%
ESR > 40 mm/h^b^	14 (50%)	14 (33%)	0.252	0.157	−9 to 43%
Platelet count > 400 000/mm^3^^c^	25 (68%)	25 (52%)	0.224	0.160	−8 to 39%

Results are presented as *n* (%). Group comparisons are made with a Mann–Whitney *U* test.

CI, confidence interval; CRP, C-reactive protein; ES, effect size; ESR, erythrocyte sedimentation rate; WBC, white blood cell.

a WBC adjusted cutoff was applied as >17 000/mL in children <48 months old for *n* = 50 hip and *n* = 49 knee.

b ESR data were available for *n* = 28 hip and *n* = 42 knee.

c Platelet count data were available for *n* = 37 hip and *n* = 48 knee.

* Level of significance was set at *P* < 0.05.

### Comparison of the weight-bearing status in septic arthritis of the hip and septic arthritis of the knee caused by *Kingella kingae*

**Fig. 4:**
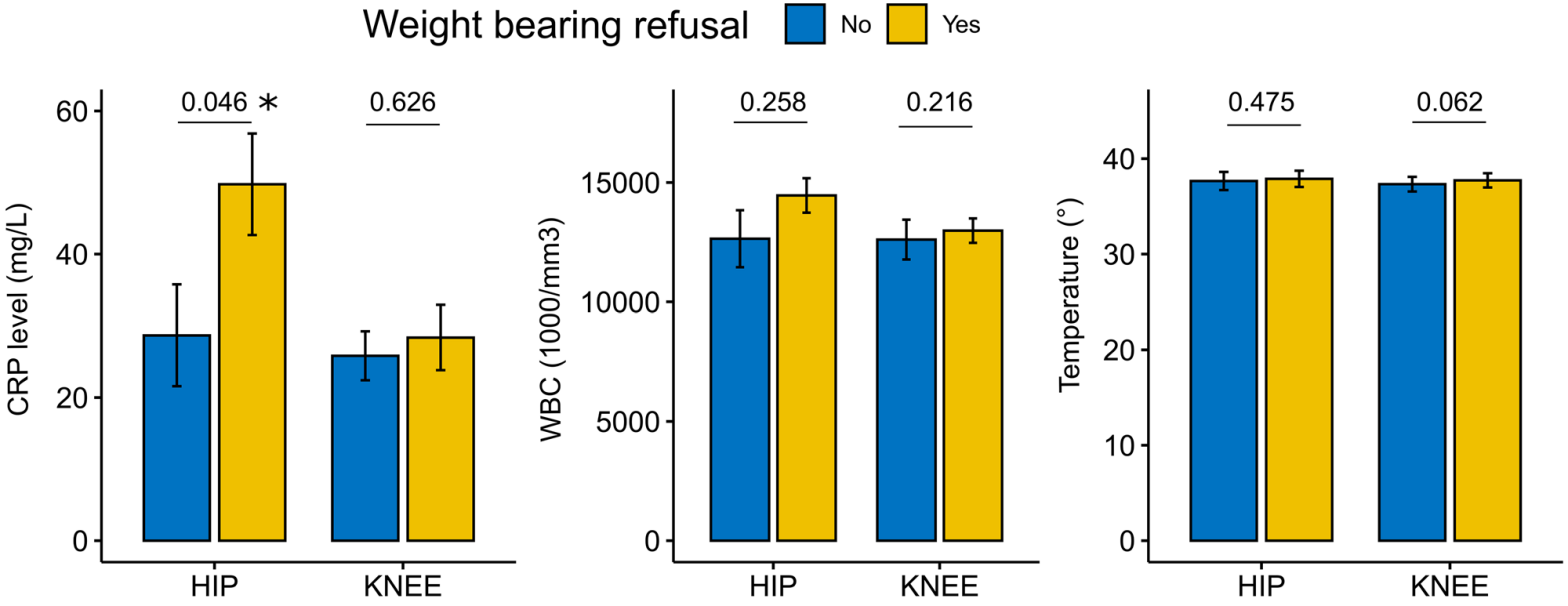
Comparison of the weight-bearing status in patients with septic arthritis of the hip and of the knee caused by *Kingella kingae* using unpaired Mann–Whitney *U* test. CRP, C-reactive protein; WBC, white blood cell.

When analyzing the weight-bearing status, the hip SA cohort encountered 30 (60%) patients who presented to the ED with weight-bearing refusal on the affected limb, against 15 (30%) patients who presented to the ED with limping but were able to bear weight on the affected limb. Five patients (10%) had no description of their weight-bearing status. In the knee SA cohort, 23 (46%) patients presented to the ED with weight-bearing refusal on the affected limb, against 27 (54%) that presented with limping but were able to bear weight on the affected limb (Fig. 4).

We tried also to identify differences in the severity of the presentation between patients with a non–weight-bearing status and patients presenting only with limping but able to bear weight.

In the hip group, CRP was statistically higher (*P* = 0.046) in the non–weight–bearing subgroup (mean: 49.8 mg/L) than in the limping subgroup (mean: 28.7 mg/L). On the contrary, we did not encounter statistically significant differences (*P* = 0.258) in the WBC between the non–weight-bearing subgroup (mean 14 458 mm^3^) and the limping subgroup (mean: 12 643 mm^3^). Temperature differences between the two subgroups were also not statistically significant (*P* = 0.475), with a mean of 37.9 °C for the non–weight-bearing subgroup and 37.7 °C for the limping subgroup.

In the knee group, CRP mean values were similar in both weight-bearing refusal subgroup (28.4 mg/L) and limping subgroup (25.8 mg/L). WBC mean values were also similar between the weight-bearing refusal subgroup (12 983 mm^3^) and the limping subgroup (12 607 mm^3^). Mean temperature did not show substantial differences between the non–weight-bearing subgroup (37.7 °C) and the limping subgroup (34.4 °C). The three values showed no statistically significant differences in the knee group between the non–weight-bearing and the limping subgroup (CRP *P = *0.626; WBC *P = *0.216; temperature *P = *0.062).

The only significant statistical difference was encountered in the CRP mean value in the hip group, this being more elevated in children presenting with a refusal to bear weight than in children presenting with a simple limping on the affected limb.

## Discussion

To the best of our knowledge, the present study represents the first attempt to investigate whether there were any significant clinical and biological differences between two different joints with SA caused by *K. kingae* in the pediatric population.

A study from Helito *et al.* [17] compared SA of the hip and knees caused by *Staphylococcus aureus* in the adult population, focusing on differences between oxacillin-resistant and nonresistant cases.

Sanpera *et al.* [18] also focused their attention on this critical subject, paying attention to the emergent diagnosis of *K. kingae* SA in children and on the reliability of the Kocher Criteria in predicting SA. They also highlighted the challenge to differentiate between Staphylococcal and *K. kingae* SA and even debated over unusual localizations other than knees and hips, such as upper limbs, in children with SA.

Our results primarily showed that the clinical presentation of SA of the hip and SA of the knee caused by *K. kingae* can differ from each other. Even if the clinical manifestations of the disease were similar in both groups (weight-bearing refusal and limping with or without temperature), we demonstrated that children with SA of the hip more frequently presented with a temperature ≥ 38.5 °C than those with SA of the knee. This observation suggests that the type of joint itself constitutes one element helping to explain how severe the clinical presentation of SA caused by *K. kingae* may be. Nevertheless, it should be noted that this work only considered large, lower-limb joints. It would thus be interesting to make comparisons with smaller joints in the future.

Despite the current unanimous recognition that the clinical picture of *K. kingae* OAIs is characterized by a mild elevation in acute-phase parameter levels, our results also showed differences in those parameters between SA of the hip and SA of the knee. Our results are the first to show significantly higher mean CRP values in *K. kingae* SA of the hip than in *K. kingae* SA of the knee. We also noted that the values for these parameters were unexpectedly dispersed.

In our experience, these differences in clinical and biological presentation do not suggest that a more severe form of the disease occurs in the hips. The higher levels of these parameters in *K. kingae* SA of the hip, however, could lead caregivers to think that they are dealing with a pyogenic infection. In our opinion, these higher levels could be explained by the diagnostic delay caused by mild clinical features at the onset of *K. kingae* SA of the hip. In a previous publication, Mallet *et al.* [19] reported on a series of unusually severe cases of *K. kingae* OAIs, particularly among those children whose diagnosis had been delayed (mean = 13.2 ± 8 days). Thus, we would legitimately expect clinical and biological parameters to be higher in this scenario.

Our study had some limitations. Its retrospective nature increased the risk of missing certain cases because of medical coding errors, and above all, it raised the proportion of missing data. Nevertheless, the descriptive material examined provided a lot of information about the clinical and laboratory presentations of children with *K. kingae* SA of the hip or the knee. Another limitation is the fact that we do not dispose of information about the delay of time between the beginning of symptoms and the hospital admission. These results should be confirmed and enriched with a future real multicenter study, which would allow us to examine a larger number of patients to define whether there are differences in terms of clinical presentation and characteristics according to the joint.

## Conclusion

This study confirmed that most of the time, both *K. kingae* SA of the hip and knee present with mild clinical and laboratory features, few suggestive of SA. Our results unexpectedly showed that *K. kingae* SA of the hip presented more frequently with a temperature ≥ 38.5 °C than did *K. kingae* SA of the knee. It also appeared that *K. kingae* SA of the hip showed significantly higher CRP values than SA of the knee. Also in the hip group, patients with a non–weight-bearing status had statistically higher CRP values than patients with simple limping. Finally, we must keep in mind that a wide range of acute-phase parameter results may be present in *K. kingae* SA.

One of the interesting aspects of our results is that CRP values seem to be higher in *K. kingae* infections around the hip and could mimic Staphylococcal infections, even if *K. kingae* has historically been known as a weak pathogen presenting with mild clinical symptoms and biological abnormalities. It appears important, in our opinion, to better understand not only the elevation of biological parameters in larger groups of patients affected by *K. kingae* SA but also to relate these biological parameters to the interval between the beginning of symptoms and the initial presentation to the ED.

It is essential, therefore, to carry out to understand whether SA affecting other joints (e.g. ankle, wrist) also have specific clinical and biological presentations.

## Acknowledgements

This project was approved by the Geneva Children’s Hospital’s Ethics Review Committee.

### Conflicts of interest

There are no conflicts of interest.
